# Love, compassion, and attachment in psychiatric care: perspectives for research and clinical practice

**DOI:** 10.47626/2237-6089-2022-0557

**Published:** 2024-04-23

**Authors:** Rodolfo Furlan Damiano, Gregory Fricchione, Euripedes Constantino Miguel

**Affiliations:** 1 Hospital das Clínicas Faculdade de Medicina Universidade de São Paulo São Paulo SP Brazil Departamento e Instituto de Psiquiatria, Hospital das Clínicas, Faculdade de Medicina, Universidade de São Paulo, São Paulo, SP, Brazil.; 2 Department of Psychiatry Massachusetts General Hospital Harvard Medical School Boston MA USA Department of Psychiatry, Massachusetts General Hospital, Harvard Medical School, Boston, MA, USA.

Modern medicine seems to be drifting away from the most important features of the clinical encounter and the benefits of clinical judgment informed by attachment, compassion, and *caritas* – the Latin word for love (i.e., humanistic traits).^1^ This risks a breakdown in patient belief in the doctor, thus undermining the physician-patient relationship.^2^ Studies have shown that trust plays a central role in attachment (i.e., “lasting psychological connectedness between human beings”)^3^ and the doctor-patient relationship.^4^ In addition, it can have a great impact on clinical outcomes^5^ related to factors like greater patient adherence.^6^ Specific functional brain changes have already been reported in secure attachment relationships in adulthood^7^ and underlying mechanisms should be further examined.

Undoubtedly, there is much debate regarding affection, attachment, and empathy in the clinical encounter, especially in psychiatric care. Discussions center on definition, structure, and practical applicability in clinical care. Perhaps the most studied of all these abstract concepts is empathy.^8^ There are now several techniques developed to improve empathy in medical students and residents. Empathy (i.e., “the cognitive attribute that involves an ability to understand the patient’s inner experiences and perspective and a capability to communicate this understanding”),^8^ in essence, is being viewed as a cognitive and behavioral trait that can be developed and taught, sometimes in a systematic way. Some studies have even pointed out that empathy (or cognitive empathy) is more important than sympathy, or affective empathy (which also might be detrimental in some cases), as the latter might be more negatively associated with emotions and affective instability.^9^ Furthermore, recent studies have attempted to understand predictors of compassion and related constructs in medical students, highlighting the importance of these constructs for physicians-to-be.^10^

The distinction between affection and cognition is more pronounced when talking about these subjective human aspects. Love and compassion, usually seen as affective virtues, have been put aside in many academic medicine settings, seen as subjective and more philosophical than scientific. This may reflect the putative struggle regarding science and religion^11^ where love, compassion and other virtues have been clustered in spiritual rather than scientific terms. When viewed as spiritual, it seems they cannot be brought into medical discussion.

When the dialogue turns to the topic of love in medicine, it behoves us to reflect back on the ancient philosophers, especially the classical Greek thinkers. In ancient Greece, there were three words to define love: *Agápe* (i.e., spiritual love), *Éros* (i.e., romantic love), and *Philia* (i.e., virtuous love among peers and community). These forms of love were described by Aristotle in Nicomachean Ethics and this is closest to the topic of our discussion here. In order to develop *Philia*, it is crucial to understand what compassion is. Compassion is related to what is known as affective empathy. It refers to the capacity to overcome some of our emotional boundaries in order to help the sufferer; since *Philia* can result in inner pain in the midst of the suffering “other,” there is an inner trait of altruism at work in this form of love. Interestingly, in Hebrew, compassion is “rechemet,” the root of which is “rechem,” which means “womb.” The womb protects the unborn child and nourishes and prepares the fetus for life. This word brings to mind the mother-infant relationship and the idea that compassion is to love like a mother.

It is also pertinent to raise an important contribution of religious tradition to this debate. In ancient times, a close connection between medicine and love/charity was absent; witness the history of physician abandonment of plague victims in the classical world. The commitment to love and charity in the practice of medicine, which can be understood as unconditional devotion to taking care of another’s needs, especially those of the poor and the sick, in the Western Christian world, is encapsulated in the concept of *caritas*. The idea of altruistic and even sacrificial care for the sick as part of the medical mission was strongly shaped by the Christian idea of charity (and by the Jesus stories), understood as unconditional love for one’s neighbor. These concepts markedly influenced the more humane response to great plagues across the historical record, where Christians, inspired by the concept of agape, altruistic love, helped unknown “siblings” and strove to reduce mortality.^12^ This helped shape the moral values of society as a whole. These ideas also influenced the creation of hospitals as charitable institutions devoted to care for the sick, with a “preferential option for the poor,” a phrase first used by South American Catholic leaders in a Church document from a 1968, meeting of the Conference of Latin American Bishops held in Medellin, Columbia.^13^ Thus, the concept of love for one’s neighbor as an essential aspect of human life (and of medical practice) has deep and lasting spiritual roots. There are of course echoes and similarities of this embedding of spiritually-based compassionate care in the effects of other religious traditions on medical practice in other cultures.^14^

Love and compassion represent a large part of attachment and the singularity of care. Ultimately, attachment represents the most important aspect of the clinical encounter. When the patient feels appreciated in all dimensions (e.g., physical, mental, spiritual), he or she will feel listened to and more deeply connected to the doctor.^15^ Treatment adherence may increase in a trustful, therapeutic environment where healing can take place, sometimes even accelerating the pace of recovery.^16^ But even when patients do not improve at the speed they (and we) want, we can accompany them, providing solace as their spirits wrestle with deep feelings and fears.^17^

So why do most physicians run away from this aspect of the healing encounter? What are our own deep emotions, fears, and boundaries? To understand this, we will need self-compassion. Overcoming our own fears of engagement could make us much more connected with our and others’ feelings and with the purpose of our profession and the core of our vocation. Physicians, including psychiatrists, do not need to run away from their inclination to be lovingly present with their patients, rather, they must acknowledge and be proud of it.

In a scientific way, we must try to understand the subjacent mechanisms of *caritas* and its impact on the medical encounter, for perhaps *caritas* enhances any medical intervention. This strategy can help to reconnect our medical science with the spiritual and subjective aspects of the human being. In this sense, virtues, positive traits, and tools to promote the patient-doctor encounter might be reconsidered as part of the therapeutic arsenal of all physicians, including psychiatrists. Spirituality (i.e., connection to something greater than ourselves and the seeking of a higher purpose) could imbue our therapeutic encounters and also be re-examined and integrated into academic medical settings.

Illness represents a separation challenge to the patient who often contemplates the potential loss of his attachments as a result of the disorder that has befallen him.^18^ The patient seeks out the healer who can find the attachment solutions that will relieve his suffering and uncertainty and return security to his attachments.^18^ Humans evolved to find a secure attachment, as love, compassion and care tend to create a safe and healing environment that fosters the development of our human potential.^19^ In developmental terms, illness can recapitulate our early childhood separation challenges leading to a sense of misattunement. In childhood, when this happens, the love of the mother is the solution for the separation fears of the child, ushering in the solace of reattunement. Throughout development, the age-appropriate separation challenges we face are solved through our healing attachments with our parents, our families, and our friends. And this will be true for the rest of our lives and relationships. When we are sick and certainly when facing the anxiety and fear of death, our patients can re-experience in the patient-doctor relationship, the healing attachment solutions that he first experienced in the *caritas* – the compassionate love – of his mother (“rechemet/rechem/womb”). Therefore, based on our brain’s evolution, we need the integration of compassionate care into whatever framework of healthcare delivery a society chooses, because compassion is physiologically, psychologically, and spiritually essential to true healing.^18^

Those who practice medicine must apply the attachment solutions that come with scientific competence at the biological level and interpersonal compassion at the humanistic level. Indeed, medicine must strive to unify the scientific and the spiritual, with both methods of relating serving as potential reflections of the attachment solutions to the separation challenges that illness presents, culminating in compassionate love at the bedside. In the final analysis, this is the only option for a medicine that professes to be humanistic.
